# Heat Shock Cognate 70 kDa Protein Is the Target of Tetradecyl 2,3-Dihydroxybenzoate for Neuritogenic Effect in PC12 Cells

**DOI:** 10.3390/biomedicines9101483

**Published:** 2021-10-16

**Authors:** Lihong Cheng, Yanhui Wang, Lan Xiang, Jianhua Qi

**Affiliations:** College of Pharmaceutical Sciences, Zhejiang University, Hangzhou 310058, China; clh83787711@zju.edu.cn (L.C.); 11519008@zju.edu.cn (Y.W.)

**Keywords:** neurodegenerative disease, Alzheimer’s disease, ABPP, target identification, PC12 cells

## Abstract

Tetradecyl 2,3-dihydroxybenzoate (ABG-001) is a lead compound derived from gentisides with a remarkable neuritogenic activity. However, the target of ABG-001 is yet to be defined to date. In this study, the potential target of ABG-001 was investigated via an activity-based protein profiling (ABPP) analysis, which is a chemical proteomic method for target identification by using chemical probes. Results indicated that the potential target proteins of ABG-001 were heat shock cognate 70 kDa protein (Hsc70), 78 kDa glucose-regulated protein (GRP78), and 14-3-3 theta protein. Then, the potential target of ABG-001 was confirmed by using inhibitors, the cellular thermal shift assay (CETSA) and small-interfering RNA (siRNA) analysis. The inhibitor of Hsc70 and siRNA significantly decreased the neurite outgrowth induced by ABG-001. Furthermore, ABG-001 induced neurite outgrowth was reduced by siRNA against Hsc70, and the results of CETSA suggested that Hsc70 showed a significant thermal stability-shifted effect upon ABG-001 treatment. These results indicated that Hsc70 is the target protein of ABG-001 in PC12 cells.

## 1. Introduction

Nerve growth factor (NGF), one of the most significant neurotrophic factors that vitally contributes to neuronal growth, survival, differentiation, and function maintenance, was once considered as an ideal therapeutic agent for the treatment of neurodegenerative diseases, such as Alzheimer’s disease (AD) [1]. However, NGF was limited as a drug candidate because of its large molecular size and hydrophilic properties that disabled it from passing through the blood–brain barrier (BBB) [2]. Accordingly, searching for compounds with considerably small molecular size and equal or even better neuritogenic activity than NGF is a possible way for neurodegenerative disease medication development. In the previous study, 11 novel alkyl benzoates (gentisides A-K) were isolated from *Gentiana rigescens* Franch, a type of traditional Chinese medicine [3,4]. According to the result of the structure-activity relationship study, several gentiside derivatives were designed and synthesized to improve neuritogenic activity. Among them, tetradecyl 2,3-dihydroxybenzoate (ABG-001) was recognized as the leading compound that exhibited optimal neuritogenic activity on PC12 cell lines [5].

ABG-001, a biologically active small molecule with excellent neuritogenic activity similar to NGF on PC12 cells, has significant potential for drug development to treat neurodegenerative diseases. Despite that ideal phenotypic screening results were obtained, the unknown mechanism of action study and target identity of the neuritogenic activity induced by ABG-001 still remained to be investigated, which are significantly crucial due to their correlations with the development of small-molecule probes and their application in biology and drug discovery [6]. In the previous study, the mechanism of action study of ABG-001 revealed that insulin growth factor 1 receptor (IGF-1R), phosphatidylinositol 3 kinase (PI3K), mitogen-activated protein kinase (MEK), and their downstream signaling cascades were involved in the neuritogenic activity on the PC12 cell [7]. However, the evidence is insufficient to conclude that IGF-1R was the target protein, and whether there are any other protein targets is yet to be determined. Therefore, performing a target-identification experiment is necessary to better understand the mechanism of action and target protein.

With the development of biological technology, proteome microarray analysis has been widely used in biological studies and clinical research because of the low reagent consumption, high sensitivity, and throughput. Microarray analysis is the basis of gene function research. Differentially expressed genes can be widely, quickly and reliably screened out for functional and pathway analysis by using microarray analysis to analyze samples at different developmental stages or under different treatment conditions [8].

The methods for target identification of small molecules from natural products mainly include designing chemical probes based on small molecules. Activity-based protein profiling (ABPP) is currently a widely used tool for target identification that utilizes probes with electrophilic groups that covalently react with enzyme active site nucleophiles [9,10]. The probes, designed according to the structure of the active leading compound, would bind to and covalently react with its target protein via an electrophilic trap or a photocrosslinking group in the living cells. A fluorophore or affinity label is attached to the complex of the target protein and covalently linked probe through the click reaction, copper-catalyzed azide-alkyne cycloaddition reaction (CuAAC) for further in-gel fluorescence analysis or MS-based proteomics [11,12]. Only those enzyme molecules with an active conformation and an exposed active site can be labeled with the probe. Therefore, some active compounds that exhibit their biological activities through their electrophilic properties are highly suitable for the ABPP approach via transforming the compound itself into the probe.

The confirmation of the target protein is also important in the process of discovery of the target of small molecules. Cellular thermal shift assay (CETSA) is an application of thermal shift assay, and it is widely used in the research of protein thermal stabilization upon ligand binding in drug discovery, at a cellular level, which is suitable for the study of target identification with a predicted target protein [13]. The procedure of the assay includes treating the cell with a candidate compound, heating to denature and precipitate proteins, cell lysis, and protein purification. Quantitative Western blotting is used to detect the thermal stability of proteins, which involves comparing the thermal stability of the predicted target protein of the treated group to that of the untreated group. The target proteins that bind with their candidate compounds would exhibit higher thermal stability or instability. A typical result of a cellular thermal shift assay shows an apparent melting curve that compares the presence or absence of the protein following the controlled temperatures in the treated and untreated groups.

In this study, the target identification of the leading compound ABG-001 was investigated via ABPP analysis. In addition, the potential target was confirmed by using the CETSA and small-interfering RNAs (siRNA) analysis. The results indicated that ABG-001 potentially targeted the Hsc70 protein.

## 2. Materials and Methods

### 2.1. Chemicals and Reagents

DMSO, NGF, Tris (2-carboxyethyl) phosphine (TCEP), tris [(1-benzyl-1H-1,2,3-triazol-4-yl) methylamine (TBTA), Cy5-azide and Azide-PEG3-biotin Conjugate were purchased from Sigma-Aldrich Co. (St. Louis, MO, USA). CuSO_4_ was purchased from Sinopharm Chemical Reagent Co. Ltd. (Shanghai, China). Pierce streptavidin magnetic beads and zeba spin desalting columns were purchased from Thermo Fisher Scientific (Waltham, MA, USA).

### 2.2. Microarray Analysis

Gene expression DNA microarray analysis was performed by LC Sciences (Houston, TX, USA) using Agilent Rat Oligo Microarray (V2), 4 × 44 K (G2519F-019161). Total RNA was extracted from PC12 cells after treatment with DMSO (negative control) or ABG-001 for 12 h by using RNA Isolation Kit (Norgen, Thorold, ON, Canada) according to the Manufacturer’s instructions, and purified by RNeasy Mini Kit (Qiagen, Hilden, Germany). RNA samples of each group were used to generate biotinylated cRNA targets and were then hybridized with the slides. After hybridization, the slides were scanned with the Agilent Microarray Scanner G5761A (Agilent Technologies, Santa Clara, CA, USA). Data were extracted with Feature Extraction software 12.0.3.1 (Agilent Technologies, Santa Clara, CA, USA). Raw data were normalized by Quantile algorithm. Genes with *p*-value < 0.05 were selected for further analysis. GO/KEGG pathway enrichment analyses were carried out using Fisher’s exact test of the target genes.

### 2.3. Preparation of ABG-001 and the Probes

ABG-001 was synthesized according to the previous report [5]. Two probes (ABG-P1 and ABG-PC) were synthesized to anchor an alkynyl handle. The detailed protocol is shown in the material and methods of the Supplementary information. The probes were purified with HPLC, and the activity of probes was measured in PC12 cells.

### 2.4. Evaluation of the Neuritogenic Activity

The PC12 cell line was purchased from the Cell Bank of the Chinese Academy of Sciences (Shanghai, China). The neuritogenic activity was evaluated as described in our previous paper [14]. Approximately, 50,000 PC12 cells were seeded in each well of a 24-well microplate and cultured under humidified conditions with 5% CO_2_ at 37 °C for 24 h. After 24 h, 1 mL serum-free Dulbecco’s modified eagle medium (DMEM) containing a test sample or DMSO (0.5%) was used to replace the previous medium in each well. NGF (40 ng/mL) was used as the positive control. Approximately 100 cells were counted thrice from a randomly selected area after 48 h treatment. Cells with neurite outgrowth longer than the diameter of their body were counted as positive cells. The percentage of the positive cells in the selected area was regarded as the activity, and the results were expressed as mean ± SEM.

In the inhibitor test, the cells in each well of 24-well microplate were first pretreated with a 500 μL culture medium containing the specific inhibitor for 30 min. Then, 500 μL of the culture medium containing the sample or DMSO was added. Morphological changes in the cells were observed after incubation of 48 h.

### 2.5. In Situ Fluorescence Labeling Using ABG-P1 and ABG-PC

PC12 cells were grown to 80–90% confluence and incubated for 24 h. Dishes supplemented with ABG-P1 or ABG-PC at the final concentration of 1 μM were incubated for specific time periods to investigate the time-dependent effects. In a dose-effect study, various concentrations of ABG-P1 were added to the dishes. Then, the dishes were incubated for a certain duration. Subsequently, the medium was removed and cells were washed with PBS and lysed with RIPA Lysis Buffer (CoWin Biosciences, Beijing, China). After centrifugation at 12,000 rpm for 15 min to obtain the supernatant, the protein concentration was detected with a BCA Protein Assay Kit (CoWin Biosciences, Beijing, China). Equal proteins (100 μg) of different treatment samples were used for the subsequent fluorescent labeling. In each reaction, Cy5-azide (1 mM), TCEP (100 mM), TBTA ligand (10 mM), and CuSO_4_ (100 mM) were added to the lysate. The samples were incubated at room temperature for 2 h. Next, clicked proteins were precipitated by acetone at −30 °C for 2 h. 1 × SDS loading buffer (50 μL) was added to dissolve the sample and was separated by sodium dodecyl sulfate polyacrylamide gel electrophoresis and visualized using a FluorChem M Multiplex fluorescence (ProteinSimple, San Jose, CA, USA). In the competition assay, the PC12 cell lysate was pretreated with ABG-001 (1 μM or 10 μM) for 12 h. Then the lysate was incubated for another 8 h together with ABG-P1 (1 μM). Probe labeled proteomes were visualized by click conjugation to the Cy5-azide tag followed by SDS-PAGE separation and fluorescence scanning. The bound proteins were collected and analyzed via mass spectrometry using LC-MS/MS (PTM Biolabs, Hangzhou, China).

### 2.6. Pull Down Assay

PC12 cells were grown to 80–90% confluence and then treated with negative control, ABG-P1, or ABG-PC for 4 h. Subsequently, the cells were collected, and the proteins were adjusted to the same amount. Equal amounts of cell lysates (5 mg) of different treatment samples were used for subsequent click chemistry to conjugate the protein with azide-PEG3-biotin separately. In each reaction, azide-PEG3-biotin (1 mM), TCEP (100 mM), TBTA ligand (10 mM), and CuSO_4_ (100 mM) were added to the lysate and incubated at room temperature for 2 h. Then, clicked proteins were subjected to the desalting column to remove the unreacted azide-PEG3-biotin. Subsequently, prewashed streptavidin magnetic beads were added to the cell lysates and incubated at 4 °C overnight. The beads were washed three times with a washing buffer (25 mM Tris, 0.15 M NaCl, and PH = 7.2) to remove the unspecific binding proteins. Finally, the beads were boiled with SDS and analyzed by Western blot.

### 2.7. Western Blot Analysis

Western blot analysis was performed in accordance with a previous study [14]. Briefly, sodium dodecyl sulfate polyacrylamide gel electrophoresis was used to separate the proteins (15 μg) and transfer them onto a PVDF membrane. Membranes were incubated with primary and secondary antibodies. Antigens were visualized using the ECL Western blot kit (Beijing CoWin Biotech Company, Beijing, China). The primary antibodies used for immunoblotting are as follows: anti-Hsc70 antibody, anti-Bip (GRP78) antibody (Abcam, Cambridge, UK), anti-IGF-1R antibody (Cell Signaling Technology, Boston, MA, USA), anti-14-3-3 theta antibody (Affinity BioReagents, OH, USA), and GAPDH antibody (Beijing Cowin Biotech Company, Beijing, China). The secondary antibodies used in this study were as follows: horseradish peroxidase-linked anti-rabbit and anti-mouse IgGs (Beijing CoWin Biotech Company, Beijing, China). Bands were quantitatively measured using ImageJ software (National Institutes of Health, Bethesda, MD, USA).

### 2.8. Cellular Thermal Shift Assay

CETSA was performed as described in other reports [13]. First, 2 × 10^6^ cells were separately added and incubated for 24 h in 60 mm dishes containing 5 mL DMEM. In each plate, ABG-001 was added at a final concentration of 1 μM. After continuous incubation for 48 h, cells were collected and heated at temperatures ranging from 50 °C to 70 °C. Finally, Western blot analysis was used to detect the change in the Hsc70, GRP78, 14-3-3 theta, and IGF-1R protein.

### 2.9. RNA Interference

PC12 cells were transfected with different concentrations of FAM-labeled siRNA to evaluate transfection efficiency. Finally, 150 nM was decided as the final concentration to perform the experiment at which 90% of transfection efficiency was obtained. The following primer sequences were used to generate siRNAs that knocked down Hsc70 and the negative control (Sangon Biotech Co. Ltd., Shanghai, China): for Hsc70, sense: 5′-GUG AAG AGC UGG AGA UGG ATT-3′, anti-sense: 5′-UCC AUC UCC AGC UCU UCA CTT-3′; for negative control, sense: 5′-UUC UCC GAA CGU GUC ACG UTT-3′, anti-sense: 5′-ACG UGA CAC GUU CGG AGA ATT-3′.

Transfection of PC12 cells with siRNA was performed on the basis of the manufacturer’s instructions. Briefly, in each well of 24-well plates, 5 × 10^4^ cells were seeded and allowed to reach 70–90% confluence in the growth medium without antibiotics one day before the transfection. Then, siRNA against Hsc70 or the negative control siRNA was used at a concentration of 150 nM with Lipofectamine 2000 (Invitrogen) as the transfection agent. After 6 h of transfection, the fresh medium containing 1 μM ABG-001 was used to replace the previous medium in the plates, and the plate was then incubated for another 24 h. Cell morphological features were observed and recorded using a microscope fitted with a camera. The percentage of the cells with neurite outgrowth was expressed as the mean ± SEM. Finally, Western blot analysis was used to detect the change in Hsc70 protein.

### 2.10. Statistical Analysis

Data were presented as mean ± SEM of three independent experiments in triplicate. Data were subjected to one-way ANOVA and Tukey’s post hoc analysis by using the GraphPad Prism software. *p* < 0.05 was considered statistically significant.

## 3. Results

### 3.1. Microarray Analytical Results after Treatment with ABG-001 in PC12 Cells

**Figure 1:**
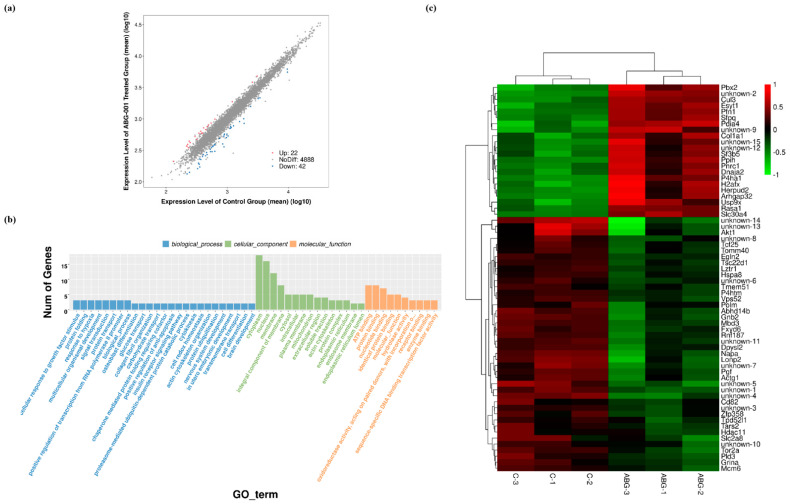
Microarray analytical results of ABG-001. (**a**) Profiles of differentially expressed mRNAs in the control group and ABG-001 treated groups. (**b**) Significant GO categories of the differentially expressed genes for the upregulated and downregulated genes. (**c**) Heatmap of differential expression genes compared with control group and ABG-001 groups. The red and green colors represent the upregulation and downregulation of genes expression, respectively.

Microarray analysis was conducted in PC12 cells after treatment with ABG-001 or negative control. The results indicated that 22 genes were up-regulated, and 42 genes were down-regulated compared with the control group (Figure 1a). The gene ontology (GO) and Kyoto Encyclopedia of Genes and Genomes pathway (KEGG) enrichment analysis showed that the involved biological processes of these genes were related to the cellular response to growth factor, cell differential, and so on (Figure 1b). It is also found that these genes participated in cellular components include cytoplasm, nucleus and mitochondrion. The heatmap of the differential expression genes is shown in Figure 1c. Among them, *P4htm* related to metal ion binding, *Fxyd6* related to ion transport, *Hspa8* related to neuron protection, *Napa* and *Akt* related to neuron differentiation were significantly down-regulated after being treated with ABG-001. Meanwhile, *P4ha1* related to metal ion binding was significantly up-regulated after being treated with ABG-001 (Figure 1c). The changes in these signaling pathways suggested that the target proteins of ABG-001 might be the key proteins in these signaling pathways. However, we need to use other methods to discover the target proteins of ABG-001.

### 3.2. Chemical Synthesis and Biological Evaluation Test of ABG-001 Based Probes in PC12 Cells

**Figure 2:**
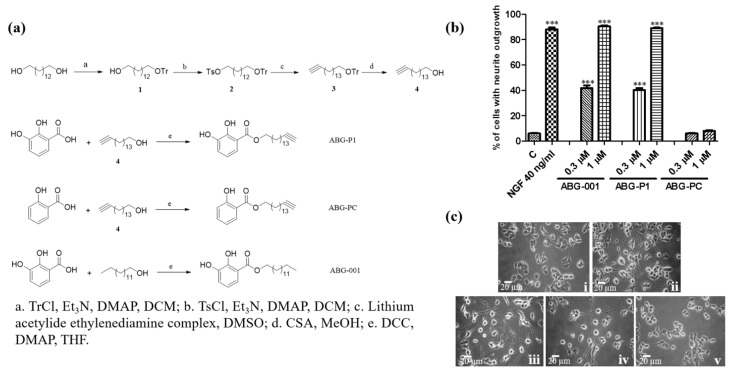
Synthesis of probes and their neurogenesis effect in PC12 cells. (**a**) Synthesis scheme and chemical structures of ABG-P1, ABG-PC, and ABG-001. (**b**) Percentage of PC12 cells with neurite outgrowth after treatment with ABG-001, ABG-P1, and ABG-PC. (**c**) Morphological changes in PC12 cells under an inverted optical microscope at 48 h after treatment with (i) control (0.5% DMSO); (ii) NGF (40 ng/mL); (iii) ABG-001 (1 μM); (iv) ABG-P1 (1 μM); (v) ABG-PC (1 μM). Scale bar, 20 μm. Each experiment was repeated thrice. The data were expressed as mean ± SEM. *** indicates significant differences at *p* < 0.001 compared with the negative control.

Probe (ABG-P1) was synthesized with an alkynyl handle (Figure 2a and Supplementary Materials and Methods). First, one of the hydroxyl groups in 1,14-tetradecanediol was protected by triphenyl methyl (Tr) to obtain compound **1** which reacted with tosyl chloride to obtain compound **2**. Then, lithium acetylide, a type of compound salt of alkynyl lithium reacted with **2** yielded compound **3**, and compound **4** was prepared by removing the triphenyl methyl group in the acidic condition. Finally, 2,3-dihydroxybenzoic acid was esterified with **4** to generate ABG-P1. We also synthesized a control probe (ABG-PC) by using salicylic acid reacted with **4** as an inactive group. The neuritogenic activities of ABG-P1 and ABG-PC were detected in PC12 cells after being treated with ABG-001, ABG-P1 and ABG-PC (1 μM) for 48 h. ABG-P1 showed a similar neuritogenic activity with ABG-001 in PC12 cells. Meanwhile, ABG-PC has no activity in PC12 cells (Figure 2b,c). Therefore, ABG-P1 and ABG-PC were used as positive probes and negative probes, respectively.

### 3.3. Target Protein Identification of ABG-001 by ABPP

**Figure 3:**
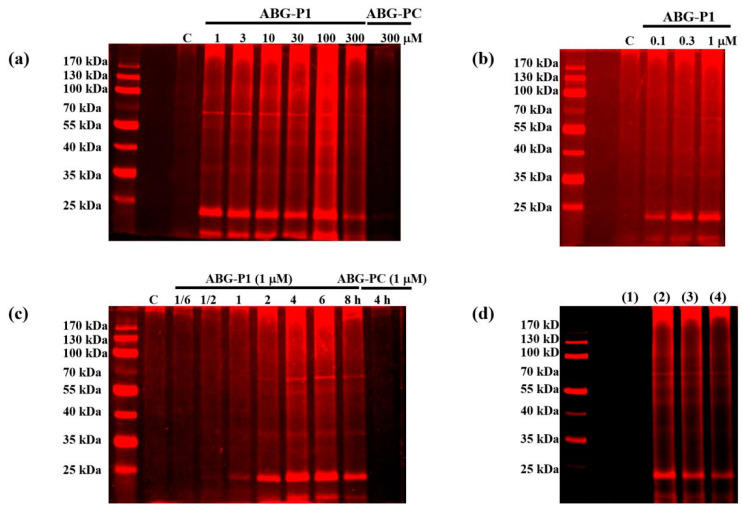
Target identification of ABG-001 by ABPP. (**a**,**b**) In situ fluorescent labeling of the PC12 cells using different concentrations of ABG-P1 and ABG-PC together with a DMSO treated negative control. (**c**) In situ fluorescent labeling of PC12 cells by treatment with ABG-P1 at different time periods. (**d**) Competition assay by pre-treating the cell lysate with ABG-001 before incubating with the ABG-P1 probes. (1) Control group; (2) ABG-P1 (100 μM); (3) ABG-001 + ABG-P1 (10 + 100 μM); (4) ABG-001 + ABG-P1(100 + 100 μM). The probe-labeled proteomes were visualized by click conjugation to the Cy5-azide tag, SDS gel separation, and fluorescent scanning.

ABPP was applied to identify the target proteins of ABG-001. Different concentrations of ABG-P1 from 0.1 μM to 300 μM were incubated with PC12 cells for 8 h. Then, the cells were lysed with lysis buffer and the lysates were then treated with Cy5-azide under click reaction (TCEP, TBTA, and CuSO_4_). Subsequently, the proteins separated by SDS-PAGE were detected via fluorescence scanning. The visible fluorescence labeling bands at 70 kDa and 25 kDa were observed, and 1 μM ABG-P1 was the optimal concentration (Figure 3a,b and Supplementary Figure S1). Then, the time-dependent experiments were conducted by treating 1 μM ABG-P1 for 1/6 to 8 h. The results demonstrated that the visible 1 μM ABG-P1 fluorescence labeling band at 70 kDa was observed at 2 h, and it arrived at its peak at 6 h (Figure 3c and Supplementary Figure S2). Furthermore, we conducted a competition assay by pre-treating the cell lysate with ABG-001 before incubating with the probes. ABG-001 pre-treatment essentially reduced the labeling signals of the probes, suggesting that the probe largely modify the same proteins as ABG-001 (Figure 3d and Supplementary Figure S1). Finally, the visible fluorescence labeling bands at 70 kDa and 25 kDa were collected and analyzed by mass spectrometry of LC-MS/MS (PTM Biolabs, Hangzhou, China). The Hsc70 and GRP78 at 70 kDa and 14-3-3 protein theta at 25 kDa had high scores in proteomic analysis (Table 1 and Table 2). These results predicted that the heat shock cognate 70 kDa protein (Hsc70), 78 kDa glucose-regulated protein (GRP78) and 14-3-3 theta protein were potential target proteins of ABG-001.

### 3.4. Target Confirmation of ABG-001 by CETSA, siRNA and Inhibitor Analysis

**Figure 4:**
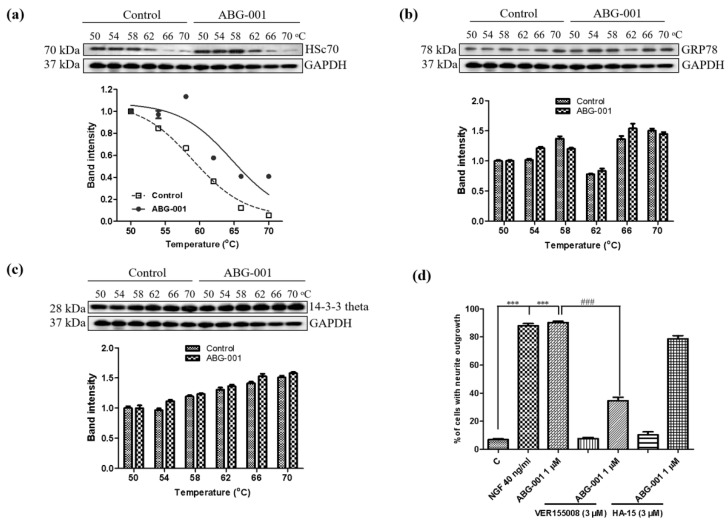
Target confirmation by CETSA and inhibitor analysis. (**a**–**c**) CETSA of PC12 cells on Hsc70, GRP78, and 14-3-3 theta protein and corresponding fitting curves. (**d**) Effect of Hsc70 (VER155008) and GRP78 (HA-15) inhibitors on the neurite outgrowth induced by ABG-001. *** indicate significant differences at *p* < 0.001 compared with the control group; ^###^ indicate significant differences at *p* < 0.001 compared with the ABG-001 treated group.

CETSA was used to discover the target protein of molecules on the basis of the thermal stabilization of proteins upon ligand binding. This mechanism was used to detect the binding correlations between the Hsc70 and the ABG-001 to further confirm the potential target of ABG-001. After treating PC12 cells with dimethyl sulfoxide (DMSO) or ABG-001 and heating at a temperature ranging from 50 °C to 70 °C, the immunoblotting analysis was conducted using a specific antibody for Hsc70, GRP78, and 14-3-3 protein theta. The markedly thermal stabilization of the Hsc70 protein upon ABG-001 treatment is shown in Figure 4a and Supplementary Figure S4a. The change in GRP78 and 14-3-3 protein theta at the protein level was detected using the same method. However, GRP78 and 14-3-3 protein theta did not show the thermal stability-shifted effect after the ABG-001 treatment (Figure 4b,c, Supplementary Figure S4b,c). These results indicated that ABG-001 might target Hsc70 to produce the NGF-mimicking activity. Furthermore, the percentage of cells with neurite outgrowth was significantly decreased from 90.3% ± 0.9% to 34.7% ± 2.4% (*p* < 0.001) after treatment with the inhibitor of Hsc70, VER155008 [15] (Figure 4d). However, the inhibitor of GRP78, HA-15 [16], did not show a significant inhibition effect (Figure 4d).

**Figure 5:**
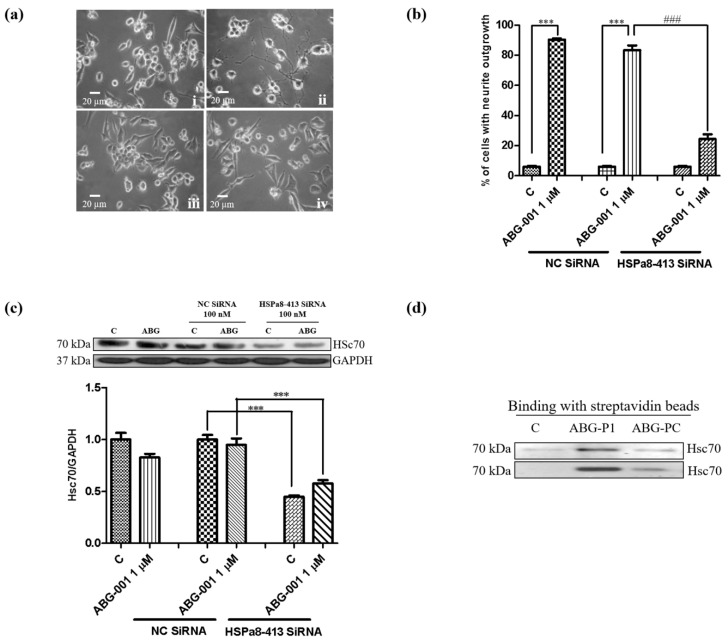
Target prediction of ABG-001 in PC12 cells by using siRNA assay and further confirmation by pull down assay. (**a**) Microphotographs of PC12 cells after treatment with siRNA and ABG-001: (i) negative control siRNA, control (0.5% DMSO); (ii) negative control siRNA, ABG-001 (1 μM); (iii) Hsc70 siRNA, control (0.5% DMSO); (iv) Hsc70 siRNA, ABG-001 (1 μM). Scale bar, 20 μm. (**b**) Percentage of cells with neurite outgrowth after treatment with siRNA and ABG-001. (**c**) Western blot analysis for Hsc70 after transfection with negative siRNA or Hsc70 siRNA and treatment with ABG-001. (**d**) The Hsc70 protein was detected by pull-down assay. ***, ^###^ indicate significant differences at *p* < 0.001 compared with the corresponding groups.

siRNA analysis was also used to make the confirmation to obtain additional evidence. The different concentrations of 5-carboxyfluorescein (FAM)-labeled siRNA (50, 100, and 150 nM) were used to validate the optimal transfection concentration of siRNA, and approximately 90% of PC12 cells produced fluorescence after treatment with 150 nM FAM-labelled siRNA. Therefore, 150 nM Hsc70 siRNA was used to perform the transfection. Hsc70 siRNA was transfected into PC12 cells for 6 h and treated with 1 μM ABG-001. After the treatment of PC12 cells with Hsc70 siRNA, the percentage of cells with neurite outgrowth induced by ABG-001 for 48 h was significantly decreased (Figure 5a,b). Besides, the total protein levels of Hsc70 were markedly decreased by the treatment with Hsc70 siRNA regardless of the ABG-001 treatment (Figure 5c, Supplementary Figure S5a). Hence, these results indicated that Hsc70 might be the target protein of ABG-001.

### 3.5. Target Identification of ABG-001 by Pull-Down Assay

A Pull-down assay was conducted to check the target protein to further confirm the target of ABG-001. PC12 cells were treated with negative control, ABG-P1, and ABG-PC for 4 h. The cell lysates were collected and applied to click reaction with biotin-azide. After unreacted biotin was removed, the cell lysates were incubated with streptavidin magnetic beads at 4 °C overnight. Subsequently, the beads were boiled with SDS to dissociate the bound protein and analyzed by Western blotting analysis. The Hsc70 protein was detected after binding with streptavidin magnetic beads in the ABG-P1 treated group. However, the Hsc70 protein was not detected in the control and ABG-PC treated groups. This experiment was repeated twice, and similar results were obtained (Figure 5d, Supplementary Figure S5b). The results showed that the same bound protein Hsc70 was identified by using the ABPP assay or pull-down assay.

## 4. Discussion

Our research group has been engaged in AD drug development for a long time. In the previous studies, we found 11 novel neuritogenic benzoate-type molecules, which are gentisides A-K from *G. rigescens* Franch under the guidance of PC12 cells bioassay system [3,4]. Their mixture was confirmed to alleviate the impaired memory of the AD model [17]. We synthesized 200 gentiside derivatives with neuritogenic effect and ascertained tetradecyl 2,3-dihydroxybenzoate (ABG-001) to be the leading compound via the structure-activity relationship study based on the chemical structure of these compounds [5]. Furthermore, ABG-001 produced not only neuritogenic effect but also anti-diabetic effect through mediating IGF-1R/PI3K/MEK signaling pathway and the insulin and adiponectin signaling pathways [7,18]. Recently, another research indicated that ABG-001 could also alleviate oligodendrocyte damage following chronic cerebral hypoperfusion through the IGF-1R signaling pathway [19]. The results of the above-mentioned studies suggested that ABG-001 is one of the drug candidates with different functions, and the target protein of ABG-001 was necessary to be identified.

In the process of drug development, target protein identification and the mechanism of action study are crucial steps that help predict the efficacy and adverse reactions of drugs. The discovery of the target protein is essential to elucidate the medicinal properties of a leading compound. The binding site conformation of the target proteins will provide additional information to design and synthesize the new leading compounds with better safety, higher activity, and better medicinal properties [20,21]. Therefore, we focused on the discovery and identification of the ABG-001 target proteins to do research work in this study. First, we used microarray analysis to predict the potential target proteins of ABG-001 from the signaling pathways that have undergone apparent changes. Then, we used the ABPP assay, pull dawn assay, LC-MS/MS analysis, CETSA assay, siRNA analysis, and inhibitor tests to confirm the target proteins of ABG-001. The experiments in Figure 1, Figure 2, Figure 3, Figure 4 and Figure 5 and Table 1 and Table 2 suggested that Hsc70 was the target protein of ABG-001 for NGF mimic effect in PC12 cells.

Hsc70, which shares its biochemical and biological properties with heat shock protein 70 (Hsp70), is an important member of the administrator in the heat shock protein A (HSPA) family [22]. The expression of Hsp70 in the brain is notable because they are highly induced in glial cells and neurons following a wide range of harmful stimuli including ischemia, epilepsy, and heat shock [23]. Hsc70 and Hsp70 were found to be significantly greater in the brains of AD patients compared with those in a normal brain [24]. Several studies indicated that overexpression of Hsp70 saved neurons from Aβ 42-mediated toxic effect in Alzheimer’s models [25,26]. Meanwhile, Hsc70 plays key roles in chaperone-mediated autophagy (CMA), regulating co-translational folding, preventing protein aggregation under stress, disassembly of clathrin-coated vesicles, and protein translocation through intracellular membranes [27,28]. Hsc70 based autophagy can effectively eliminate Aβ oligomers and has superior neuroprotective activity [29]. This evidence indicated that Hsc70 is an important protein in neurodegenerative disease. Therefore, we will investigate whether ABG-001 targets Hsc70 to improve the memory of AD mice at the animal level in the near future.

In our previous study, we found that the IGF-1 signaling pathway played an important role in the NGF mimic effect in PC12 cells. We speculated that the IFG-1 receptor was one of the target proteins of ABG-001. In this study, we used CETSA and Western blotting to confirm this notion. The result in Supplementary Figure S3 indicated that the IFG-1 receptor was not the target protein of ABG-001. In addition, some natural active compounds, such as CuB, amarogentin, 3beta, 23, 28-trihydroxy-12-oleanene 3beta-caffeate from the traditional Chinese medicines (TCMs) target cofilin, insulin receptor and ER to produce neuritogenic activity, respectively [14,30,31]. Therefore, the correlation of these target proteins will be studied in the future.

In conclusion, Hsc70 was identified to be the target protein of the leading compound ABG-001 which was derived from neuritogenic gentisides. However, the binding site between the Hsc70 protein and ABG-001 and whether ABG-001 targets Hsc70 to improve the memory of AD mice at the animal level needs to be investigated in the future. These studies will provide substantial information for AD drug development of ABG-001.

## Figures and Tables

**Table 1:** Protein identification results of the 70 kDa band in Figure 3.

	Protein Names	Gene Names	MW [kDa]	Protein Score	Sequence Coverage (%)	Unique Peptides	Peptides	PSMs
P63018	Heat shock cognate 71 kDa protein (Hsc70)	Hspa8	70.83	2904.42	37.15	20	24	70
P06761	Endoplasmic reticulum chaperone BiP (GRP78)	Hspa5	72.30	2197.30	38.07	21	23	48
F1M953	Stress-70 protein, mitochondrial	Hspa9	73.70	924.84	34.90	18	18	21
G3V8L3	Lamin A, isoform CRA_b	Lmna	74.27	837.03	29.77	16	16	19
Q9EPH8	Polyadenylate-binding protein 1	Pabpc1	70.66	717.99	28.30	13	16	17

**Table 2:** Protein identification results of the 25 kDa band in Figure 3.

	Protein Names	Gene Names	MW [kDa]	Protein Score	Sequence Coverage (%)	Unique Peptides	Peptides	PSMs
P68255	14-3-3 protein theta	Ywhaq	27.76	693.16	33.47	7	9	15
G3V913	Heat shock 27 kDa protein 1	Hspb1	22.79	495.84	36.89	7	7	12
P68511	14-3-3 protein eta	Ywhah	28.19	468.72	29.67	6	8	11
P63102	14-3-3 protein zeta/delta	Ywhaz	27.75	449.39	29.8	6	8	13
P35213	14-3-3 protein beta/alpha	Ywhab	28.04	439.91	29.67	4	6	10

## Data Availability

All figures and data used to support this study are included in this article.

## Supplementary Materials

The following are available online at https://www.mdpi.com/article/10.3390/biomedicines9101483/s1. Figure S1: The in situ fluorescent labeling of PC12 cells using different concentrations of ABG-P1 and ABG-PC together with a DMSO treated negative control and corresponding Coomassie staining results; Figure S2: The in situ fluorescent labeling of PC12 cells by treated with ABG-P1 at the different time period and corresponding Coomassie staining results; Figure S3: CETSA of PC12 cells on IGF-1R and corresponding fitting curves; Figures S4 and S5: Origin data of Western blot analysis in Figure 4 and Figure 5.

## Abbreviations

ABPP, activity-based protein profiling; AD, Alzheimer’s disease; BBB, blood–brain barrier; cellular thermal shift assay (CETSA); GRP78, 78-kDa glucose-regulated protein; IGF-1R, insulin-like growth factor 1 receptor; MEK, mitogen-activated protein kinase; PD, Parkinson’s disease; PI3K, phosphatidylinositol 3 kinase; NGF, nerve growth factor; small-interfering RNA (siRNA).

## References

[1] Xu C.J., Wang J.L., Jin W.L. (2016). The emerging therapeutic role of NGF in Alzheimer’s Disease. Neurochem. Res..

[2] Aloe L., Rocco M.L., Balzamino B.O., Micera A. (2015). Nerve growth factor: A focus on neuroscience and therapy. Curr. Neuropharmacol..

[3] Gao L.J., Li J.Y., Qi J.H. (2010). Gentisides A and B, two new neuritogenic compounds from the traditional Chinese medicine *Gentiana rigescens* Franch. Bioorg. Med. Chem..

[4] Gao L.J., Xiang L., Luo Y., Wang G.F., Li J.Y., Qi J.H. (2010). Gentisides C-K: Nine new neuritogenic compounds from the traditional Chinese medicine *Gentiana rigescens* Franch. Bioorg. Med. Chem..

[5] Luo Y., Sun K.Y., Li L., Gao L.J., Wang G.F., Qu Y., Xiang L., Chen L., Hu Y.Z., Qi J. (2011). Structure-activity relationships of neuritogenic Gentiside derivatives. ChemMedChem.

[6] Danese S., Fiocchi C., Panes J. (2016). Drug development in IBD: From novel target identification to early clinical trials. Gut.

[7] Tang R.Q., Gao L.J., Kawatani M., Chen J.Z., Cao X.L., Osada H., Xiang L., Qi J. (2015). Neuritogenic activity of tetradecyl 2,3-dihydroxybenzoate is mediated through the insulin-like growth factor 1 receptor/phosphatidylinositol 3 kinase/mitogen-activated protein kinase signaling pathway. Mol. Pharmacol..

[8] Qi H., Wang F., Tao S. (2019). Proteome microarray technology and application: Higher, wider, and deeper. Expert Rev. Proteomics.

[9] Cravatt B.F., Wright A.T., Kozarich J.W. (2008). Activity-based protein profiling: From enzyme chemistry. Annu. Rev. Biochem..

[10] Fonovic M., Bogyo M. (2008). Activity-based probes as a tool for functional proteomic analysis of proteases. Expert Rev. Proteomics.

[11] Tornoe C.W., Christensen C., Meldal M. (2002). Peptidotriazoles on solid phase: [1,2,3]-triazoles by regiospecific copper(I)-catalyzed 1,3-dipolar cycloadditions of terminal alkynes to azides. J. Org. Chem..

[12] Rostovtsev V.V., Green L.G., Fokin V.V., Sharpless K.B. (2002). A stepwise Huisgen cycloaddition process: Copper(I)-catalyzed regioselective “ligation” of azides and terminal alkynes. Angew. Chem. Int. Ed. Engl..

[13] Jafari R., Almqvist H., Axelsson H., Ignatushchenko M., Lundback T., Nordlund P., Molina D.M. (2014). The cellular thermal shift assay for evaluating drug target interactions in cells. Nat. Protoc..

[14] Cheng L.H., Osada H., Xing T.Y., Yoshida M., Xiang L., Qi J. (2021). The insulin receptor: A potential target of amarogentin isolated from *Gentiana rigescens* Franch that induces neurogenesis in PC12 cells. Biomedicines.

[15] Schlecht R., Scholz S.R., Dahmen H., Wegener A., Sirrenberg C., Musil D., Bomke J., Eggenweiler H.M., Mayer M.P., Bukau B. (2013). Functional analysis of Hsp70 inhibitors. PLoS ONE.

[16] Cerezo M., Lehraiki A., Millet A., Rouaud F., Plaisant M., Jaune E., Botton T., Ronco C., Abbe P., Amdouni H. (2016). Compounds triggering ER stress exert anti-melanoma effects and overcome BRAF inhibitor resistance. Cancer Cell.

[17] Li J., Gao L.J., Sun K.Y., Xiao D., Li W.Y., Xiang L., Qi J.H. (2016). Benzoate fraction from *Gentiana rigescens* Franch alleviates scopolamine induced impaired memory in mice model in vivo. J. Ethnopharmacol..

[18] Xiang L., Li J., Wang Y.H., Tang R.Q., Wang Q., Wu Q.B., Qi J.H. (2017). Tetradecyl 2,3-dihydroxybenzoate improves the symptoms of diabetic mice by modulation of insulin and adiponectin signaling pathways. Front. Pharmacol..

[19] Youssef M.I., Zhou Y., Eissa I.H., Wang Y., Zhang J., Jiang L., Hu W., Qi J., Chen Z. (2020). Tetradecyl 2,3-dihydroxybenzoate alleviates oligodendrocyte damage following chronic cerebral hypoperfusion through IGF-1 receptor. Neurochem. Int..

[20] Liu Y., Lv S., Peng L., Xie C., Gao L., Sun H., Lin L., Ding K., Li Z. (2021). Development and application of novel electrophilic warheads in target identification and drug discovery. Biochem. Pharmacol..

[21] Ha J., Park H., Park J., Park S.B. (2021). Recent advances in identifying protein targets in drug discovery. Cell Chem. Biol..

[22] Stricher F., Macri C., Ruff M., Muller S. (2013). HSPA8/HSC70 chaperone protein: Structure, function, and chemical targeting. Autophagy.

[23] Franklin T.B., Krueger-Naug A.M., Clarke D.B., Arrigo A.P., Currie R.W. (2005). The role of heat shock proteins Hsp70 and Hsp27 in cellular protection of the central nervous system. Int. J. Hyperth..

[24] Piedrahita D., Castro-Alvarez J.F., Boudreau R.L., Villegas-Lanau A., Kosik K.S., Gallego-Gomez J.C., Cardona-Gomez G.P. (2016). β-Secretase 1’s targeting reduces hyperphosphorilated tau, implying autophagy actors in 3xTg-AD mice. Front. Cell. Neurosci..

[25] Magrane J., Smith R.C., Walsh K., Querfurth H.W. (2004). Heat shock protein 70 participates in the neuroprotective response to intracellularly expressed β-amyloid in neurons. J. Neurosci..

[26] Smith R.C., Rosen K.M., Pola R., Magrané J. (2005). Stress proteins in Alzheimer’s disease. Int. J. Hyperth..

[27] Cuervo A.M., Dice J.F. (2000). Age-related decline in chaperone-mediated autophagy. J. Biol. Chem..

[28] Massey A.C., Kaushik S., Sovak G., Kiffin R., Cuervo A.M. (2006). Consequences of the selective blockage of chaperone-mediated autophagy. Proc. Natl. Acad. Sci. USA.

[29] Dou J., Su P., Xu C., Wen Z., Mao Z., Li W. (2020). Targeting Hsc70-based autophagy to eliminate amyloid β oligomers. Biochem. Biophys. Res. Commun..

[30] Cheng L.H., Muroi M., Cao S.N., Bian L.L., Osada H., Xiang L., Qi J.H. (2019). 3β,23,28-Trihydroxy-12-oleanene 3β-Caffeate from *Desmodium sambuense*-induced neurogenesis in PC12 cells mediated by ER stress and BDNF-TrkB signaling pathways. Mol. Pharm..

[31] Li J., Sun K.Y., Muroi M., Gao L.J., Chang Y.T., Osada H., Xiang L., Qi J.H. (2019). Cucurbitacin B induces neurogenesis in PC12 cells and protects memory in APP/PS1 mice. J. Cell. Mol. Med..

