# Supplementary material for: Heat Shock Cognate 70 kDa Protein Is the Target of Tetradecyl 2,3-Dihydroxybenzoate for Neuritogenic Effect in PC12 Cells
Source: Biomedicines. 2021 Oct 16;9(10):1483. doi: 10.3390/biomedicines9101483 (PMC8533567; doi:10.3390/biomedicines9101483)
Supplement: Supplementary file 1 [file biomedicines-09-01483-s001.zip › biomedicines-1393505-supplementary.pdf]

Materials and Methods

1. Synthesis

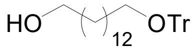

1

1,14-tetradecanediol (230 mg, 1.0 mmol) was dissolved in 10 mL anhydrous dichloromethane. Then triethylamine (210 μL, 1.5 mmol) and DMAP (12 mg, 0.1 mmol) were added. The TrCl (279 mg, 1.0 mmol) was then added in batches under ice bath which was stirred overnight at room temperature. The 1 mL methanol was added to quench the reaction and the reaction mixture was dissolved in CH_2Cl_2, washed with saturated salt water (3×40 mL). The organic layer was dried with anhydrous sodium sulfate and concentrated. The colorless powder (**1**, 180 mg, 38%) was obtained after silica gel open column purification (*n*-hexane: ethyl acetate = 10:1).

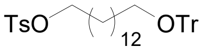

2

Compound **1** (180 mg, 0.382 mmol) was dissolved in 10 mL anhydrous dichloromethane. The triethylamine (108 μL, 0.774 mmol) and DMAP (5 mg, 0.04 mmol) were added. Then TsCl (81 mg, 0.426 mmol) was added in batches under ice bath which stirred overnight at room temperature. The 1 mL methanol was added to quench the reaction and the reaction mixture was dissolved in CH_2Cl_2, and washed with saturated salt water (3×40 mL). The organic layer was dried with anhydrous sodium sulfate and concentrated. The colorless powder (**2**, 200 mg, 83%) was obtained by silica gel open column purification (*n*-hexane: ethyl acetate = 20:1). ^{1}H NMR (500 MHz, $CDCl_3$): δ 7.79 (d, 2H, *J*=8.2Hz), 7.46-7.21 (m, 17H), 4.02 (t, 2H, *J*=6.5 Hz), 3.05 (t, 2H, *J*=6.5 Hz), 2.45 (s, 3H), 1.66-1.59 (m, 4H), 1.36-1.21 (m, 20H).

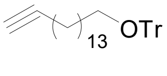

3

Compound **2** (200 mg, 0.31 mmol) was dissolved in 2 mL anhydrous DMSO. The Lithium acetylide ethylenediamine complex (90%, 70 mg, 0.68 mmol) was added. The

reaction mixture was stirred overnight at room temperature. Then 100 mL CH_2Cl_2 was added to dilute the reaction mixture. The organic layer was washed with saturated salt water (3×40 mL), dried with anhydrous sodium sulfate and concentrated. The colorless powder (**3**, 75 mg, 50%) was obtained by silica gel open column purification (*n*-hexane: ethyl acetate = 100:1-50:1). ^{1}H NMR (500 MHz, $CDCl_3$): δ 7.46-7.44 (m, 6H), 7.31-7.21 (m, 9H), 3.04 (t, 2H, *J*=6.5 Hz), 2.20-2.16 (m, 2H), 1.94 (t, 1H, *J*=2.5 Hz), 1.64-1.59 (m, 2H), 1.54-1.50 (m, 2H), 1.41-1.25 (m, 20H).

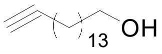

4

Compound **3** (75 mg, 0.15 mmol) was dissolved in 20 mL mixture of DCM and CH_3OH (1:1). The camphorsulfonic acid (3.5 mg, 0.015 mmol) was added, then reaction mixture was stirred at room temperature. Two hours later 1 mL of triethylamine was added to quench the reaction. The colorless powder (**4**, 4 mg, 81%) was obtained by silica gel open column purification (*n*-hexane: ethyl acetate = 20:1). ^{1}H NMR (500 MHz, $CDCl_3$): δ 3.64 (t, 2H, *J*=6.5 Hz), 2.19-2.16 (m, 2H), 1.93 (t, 1H, *J*=2.5 Hz), 1.59-1.49 (m, 4H), 1.40-1.26 (m, 20H).

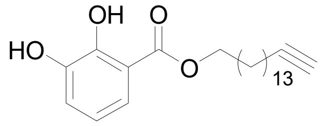

5 (ABG-P1)

Compound **4** (30 mg, 0.143 mmol) was dissolved in 5 mL dried dichloromethane. The DCC (59 mg, 0.286 mmol) and 2,3-dihydroxybenzoic acid (33 mg, 0.215 mmol) were added and reacted overnight at room temperature. Then the reaction mixture was filtered and the filtrate was concentrated. Finally, a colorless powder (**5**, 23 mg, 46%) was obtained after silica gel open column purification (*n*-hexane: ethyl acetate = 10:1). ^{1}H NMR (500 MHz, $CDCl_3$): δ 11.00 (s, 1H), 7.37 (dd, 1H, *J*=8.0, 1.2 Hz), 7.10 (dd, 1H, *J*=7.8, 1.2 Hz), 6.80 (t, 1H, *J*=8.0 Hz), 5.67 (s, 1H), 4.34 (t, 2H, *J*=6.7 Hz), 2.19-2.16 (m, 2H), 1.94 (t, 1H, *J*=2.5 Hz), 1.80-1.75 (m, 2H), 1.55-1.49 (m, 2H), 1.45-1.28 (m, 20H). HRMS (ESI) *m/z* calcd for $C_{23}H_{34}O_4Na^+$ $[M + Na]^+$ 397.2349, Found 397.2350.

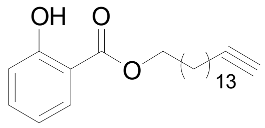

6 (ABG-PC)

Compound **4** (30 mg, 0.143 mmol) was dissolved in 5 mL dried dichloromethane. The DCC (59 mg, 0.286 mmol) and salicylic acid (30 mg, 0.215 mmol) were added and reacted overnight at room temperature. Then the reaction mixture was filtered and concentrated. Finally, a colorless powder (**6**, 20 mg, 45%) was obtained by silica gel open column purification (*n*-hexane: ethyl acetate = 10:1). ^{1}H NMR (500 MHz, $CDCl_3$): δ 10.85 (s, 1H), 7.84 (dd, 1H, *J*=8.0, 1.3 Hz), 7.45 (t, 1H, *J*=8.0 Hz), 6.98 (dd, 1H, *J*=8.0, 1.3 Hz), 6.88 (t, 1H, *J*=8.0 Hz), 5.66 (s, 1H), 4.34 (t, 2H, *J*=6.7 Hz), 2.19-2.16 (m, 2H), 1.94 (t, 1H, *J*=2.5 Hz), 1.78-1.75 (m, 2H), 1.55-1.49 (m, 2H), 1.46-1.26 (m, 20H).

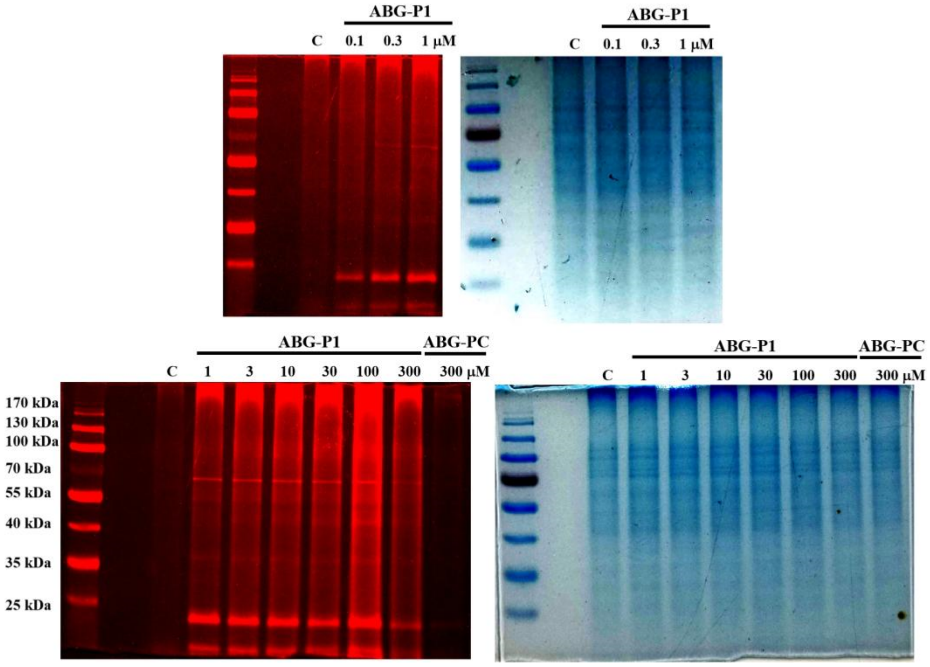

Supplementary Figure 1. The in situ fluorescent labelling of PC12 cells using different concentrations of ABG-P1 and ABG-PC together with a DMSO treated negative control and corresponding coomassie staining results.

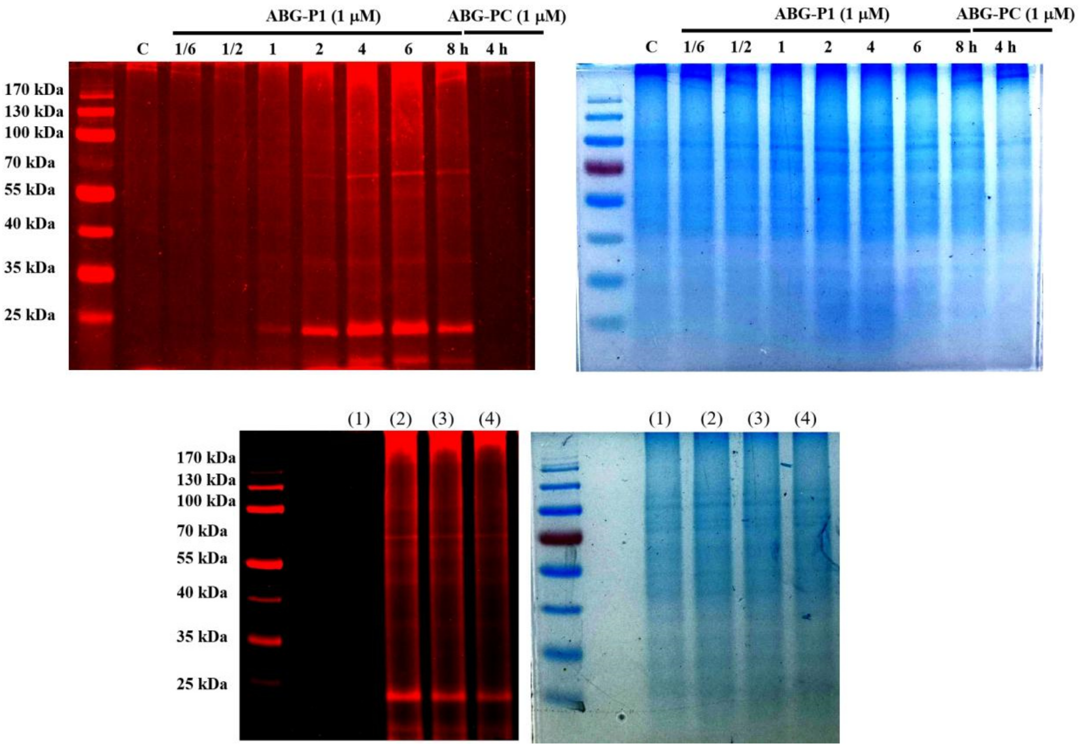

Supplementary Figure 2. The in situ fluorescent labelling of PC12 cells by treated with ABG-P1 at different time period and corresponding coomassie staining results.

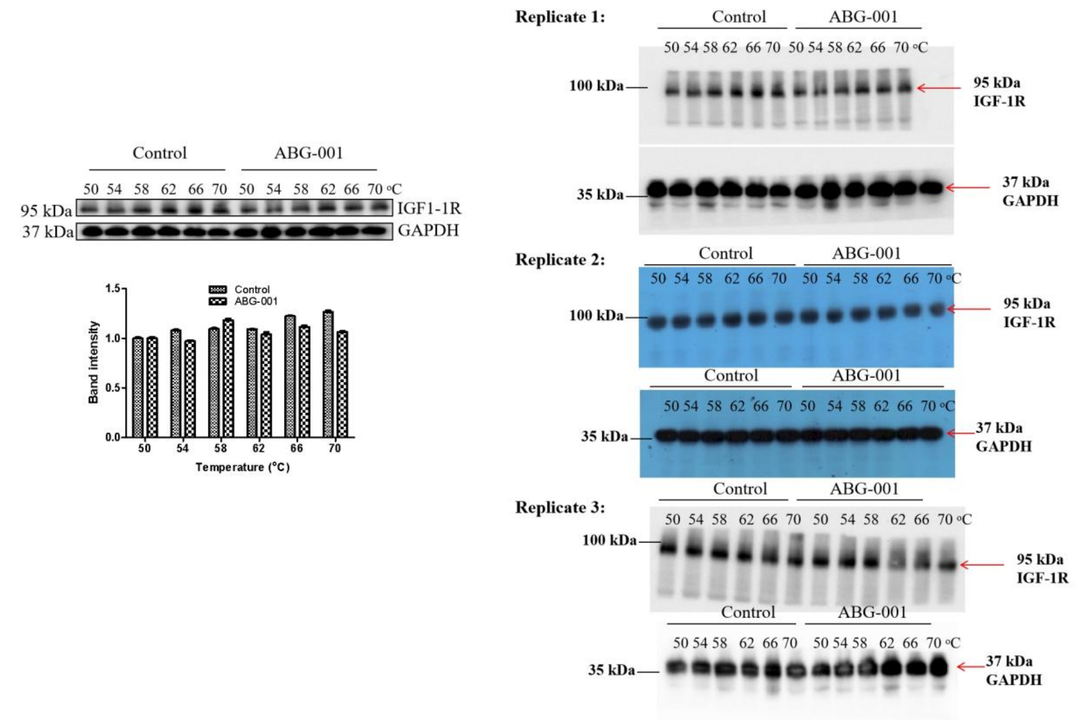

Supplementary Figure 3. CETSA of PC12 cells on IGF-1R and corresponding fitting curves.

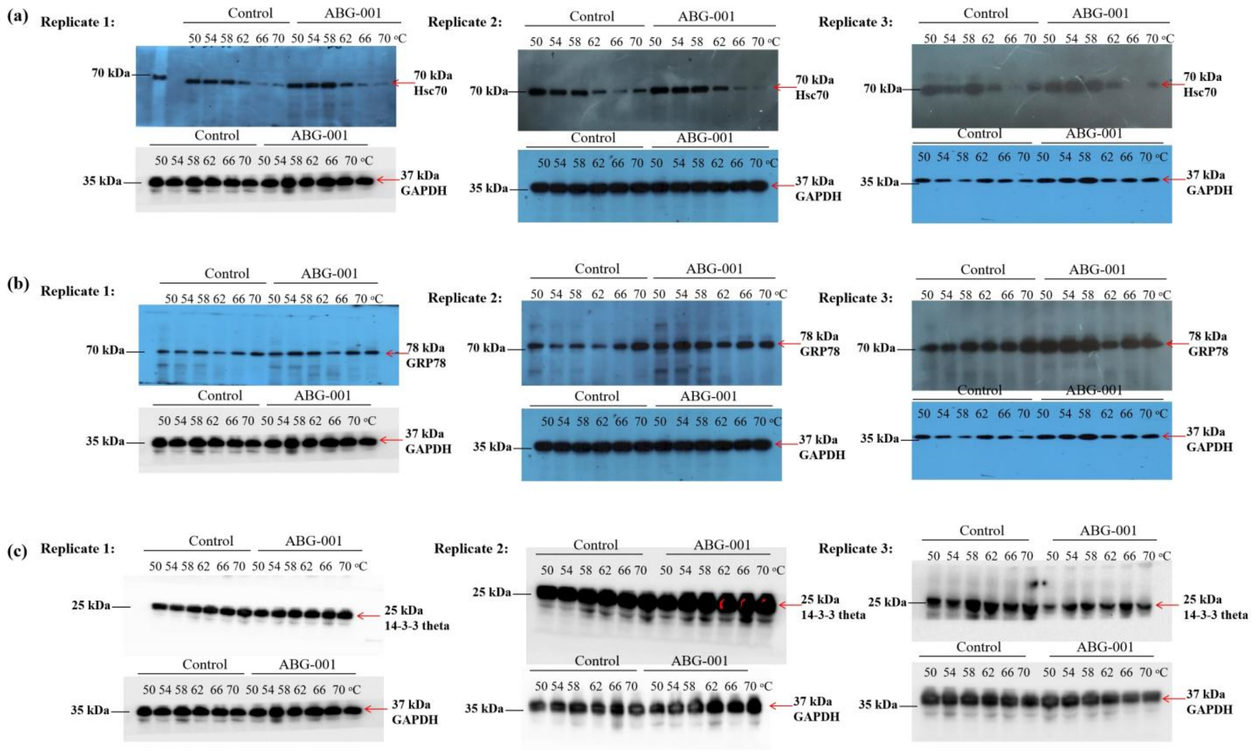

Supplementary Figure 4. Origin data of Western blot analysis in Figure 4.

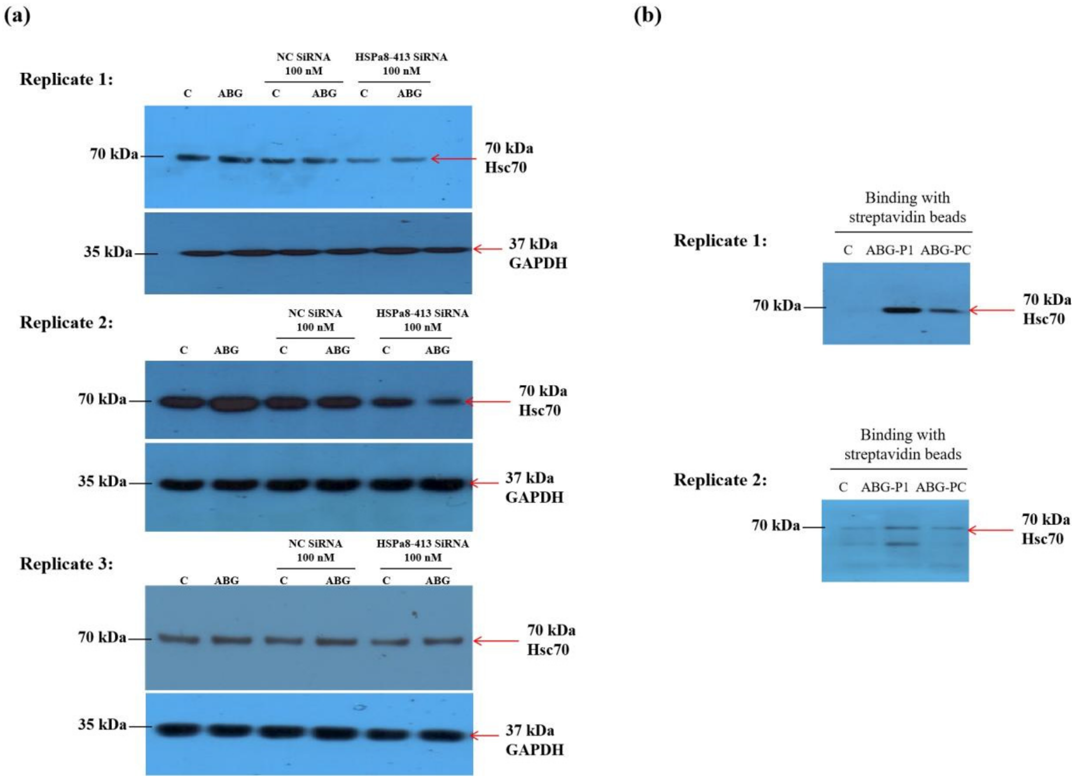

Supplementary Figure 5. Origin data of Western blot analysis in Figure 5.
